# P-1963. Signal Detection of Antibiotic-Associated Hematologic Adverse Events: Aplastic Anemia, Agranulocytosis, and Pancytopenia in the FAERS Database

**DOI:** 10.1093/ofid/ofaf695.2130

**Published:** 2026-01-11

**Authors:** Dona Ann Varkey, Jose J Kochuparambil

**Affiliations:** Mary Queens Mission Hospital, Anakkallu, Kerala, India; MQMH, Pala, Kerala, India

## Abstract

**Background:**

Antibiotics are among the most commonly prescribed drug classes worldwide. While generally safe, several antibiotics have been implicated in rare but life-threatening hematologic adverse events (AEs), including aplastic anemia, agranulocytosis, and pancytopenia. These events are often underrecognized due to their delayed onset and non-specific clinical presentation. This study aimed to identify signal strength and antibiotic associations for serious hematologic AEs using post-marketing data from the U.S. FDA Adverse Event Reporting System (FAERS).

**Figure:**
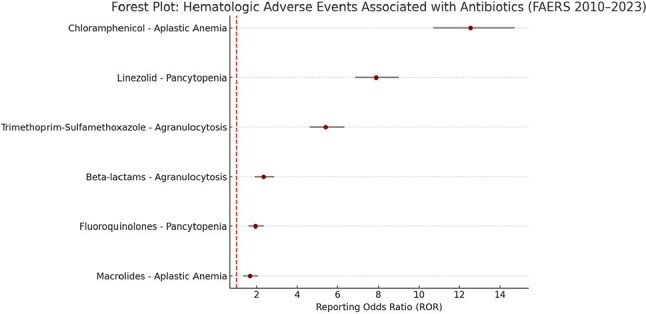
Forest Plot: Hematologic Adverse Events Associated with Antibiotics (FAERS 2010–2023) This forest plot presents Reporting Odds Ratios (RORs) with 95% confidence intervals for antibiotic-associated hematologic adverse events. Chloramphenicol showed the strongest signal for aplastic anemia (ROR: 12.56), followed by linezolid for pancytopenia (ROR: 7.88) and trimethoprim-sulfamethoxazole for agranulocytosis (ROR: 5.41). These findings reinforce the need for routine hematologic monitoring during therapy with high-risk antibiotics.

**Methods:**

A retrospective pharmacovigilance analysis was conducted using FAERS data from January 2010 to December 2023. Reports listing commonly used antibiotics (e.g., chloramphenicol, linezolid, trimethoprim-sulfamethoxazole, beta-lactams, fluoroquinolones, and macrolides) were screened for hematologic-related MedDRA Preferred Terms including “aplastic anemia,” “agranulocytosis,” and “pancytopenia.” Disproportionality analysis was performed using Reporting Odds Ratios (ROR) and 95% confidence intervals (CI). A signal was considered significant if the lower bound of the CI >1 and ≥3 reports existed.

**Results:**

Out of 5,284 relevant hematologic AE reports, the highest signals were detected for chloramphenicol and aplastic anemia (ROR: 12.56, 95% CI: 10.71–14.73), followed by linezolid and pancytopenia (ROR: 7.88), and trimethoprim-sulfamethoxazole and agranulocytosis (ROR: 5.41). Beta-lactams and fluoroquinolones showed lower but statistically significant signals. Most events occurred within 2–4 weeks of antibiotic initiation, and approximately 21.6% required hospitalization or resulted in life-threatening outcomes.

**Conclusion:**

This FAERS-based analysis reveals strong and class-specific signals for hematologic toxicity linked to antibiotics. Chloramphenicol, linezolid, and sulfonamides demonstrated the highest risk. Clinicians should maintain vigilance for hematologic monitoring in prolonged or high-dose therapy. These findings support ongoing pharmacovigilance and the inclusion of hematologic safety warnings in prescribing guidelines.

**Disclosures:**

All Authors: No reported disclosures

